# HIV-1 with Multiple CCR5/CXCR4 Chimeric Receptor Use Is Predictive of Immunological Failure in Infected Children

**DOI:** 10.1371/journal.pone.0003292

**Published:** 2008-09-29

**Authors:** Mariangela Cavarelli, Ingrid Karlsson, Marisa Zanchetta, Liselotte Antonsson, Anna Plebani, Carlo Giaquinto, Eva Maria Fenyö, Anita De Rossi, Gabriella Scarlatti

**Affiliations:** 1 Viral Evolution and Transmission Unit, DIBIT, Fondazione Centro San Raffaele, Milan, Italy; 2 Division of Medical Microbiology/Virology, Department of Laboratory Medicine, Lund University, Lund, Sweden; 3 Department of Oncology and Surgical Sciences, Unit of Viral Oncology, AIDS Reference Center, University of Padova, IOV-IRCCS, Padova, Italy; 4 Division of Cellular and Molecular Pharmacology, Department of Experimental Medical Science, Lund University, Lund, Sweden; 5 Department of Pediatrics, University of Milan, Clinica De Marchi, Milan, Italy; 6 Department of Pediatrics, University of Padova, Padova, Italy; AIDS Research Center, Chinese Academy of Medical Sciences and Peking Union Medical College, China

## Abstract

**Background:**

HIV-1 R5 viruses are characterized by a large phenotypic variation, that is reflected by the mode of coreceptor use. The ability of R5 HIV-1 to infect target cells expressing chimeric receptors between CCR5 and CXCR4 (R5^broad^ viruses), was shown to correlate with disease stage in HIV-1 infected adults. Here, we ask the question whether phenotypic variation of R5 viruses could play a role also in mother-to-child transmission (MTCT) of HIV-1 and pediatric disease progression.

**Methodology/Principal Findings:**

Viral isolates obtained from a total of 59 HIV-1 seropositive women (24 transmitting and 35 non transmitting) and 28 infected newborn children, were used to infect U87.CD4 cells expressing wild type or six different CCR5/CXCR4 chimeric receptors. HIV-1 isolates obtained from newborn infants had predominantly R5^narrow^ phenotype (n = 20), but R5^broad^ and R5X4 viruses were also found in seven and one case, respectively. The presence of R5^broad^ and R5X4 phenotypes correlated significantly with a severe decline of the CD4+ T cells (CDC stage 3) or death within 2 years of age. Forty-three percent of the maternal R5 isolates displayed an R5^broad^ phenotype, however, the presence of the R5^broad^ virus was not predictive for MTCT of HIV-1. Of interest, while only 1 of 5 mothers with an R5X4 virus transmitted the dualtropic virus, 5 of 6 mothers carrying R5^broad^ viruses transmitted viruses with a similar broad chimeric coreceptor usage. Thus, the maternal R5^broad^ phenotype was largely preserved during transmission and could be predictive of the phenotype of the newborn's viral variant.

**Conclusions/Significance:**

Our results show that R5^broad^ viruses are not hampered in transmission. When transmitted, immunological failure occurs earlier than in children infected with HIV-1 of R5^narrow^ phenotype. We believe that this finding is of utmost relevance for therapeutic interventions in pediatric HIV-1 infection.

## Introduction

Mother-to-child transmission (MTCT) of human immunodeficiency virus type 1 (HIV-1) is the primary mode of infection in children. In year 2007, an estimated 420.000 new infections occurred in children aged less than 15 years, most living in Sub-Saharan Africa [1].

Several maternal parameters, including advanced clinical stage, low CD4+ T cell counts, high plasma viral load were associated with an increased risk of MTCT of HIV-1 (reviewed in [2]). There are controversial data concerning the role of viral phenotype in transmission. Although viruses using CXCR4 as coreceptor (X4 phenotype) can be transmitted when present in the mother, CCR5-using viruses (R5 phenotype) are the most frequently detected in newborns [3], [4].

Evolution of HIV-1 coreceptor use during disease progression has been demonstrated in adults as well as children [5], [6]. The evolution usually involves change from CCR5 use (R5 phenotype) to CXCR4 use alone (X4) or in combination with CCR5 (R5X4) and/or other minor coreceptors (multitropic viruses). CXCR4-using viruses can be isolated prior to or during progression to AIDS, however only from about one-half of patients with overt AIDS [7], [8], thus suggesting that R5 viruses obtained during clinical progression may differ in phenotypic characteristics from those obtained during the early stages of infection.

Phenotypic variation characterizes R5 viruses, as demonstrated by their varying capacity to infect macrophages [9], [10], [11], [12] or their differential susceptibility to inhibition by CC-chemokines [6], [13], [14]. Studies on the entry of R5 viruses into cells expressing CCR5/CXCR4 chimeric receptors [15], [16] showed that the differential susceptibility to inhibition by CC-chemokines depends on the mode of CCR5 use. In particular, it has been shown that during disease progression R5 viruses evolve to multiple chimeric receptor usage (called R5^broad^), which in turn correlated with CD4+ T cell decline in the patient [15]. Evolution of the R5^broad^ phenotype was associated with decreased sensitivity to inhibition by the CC-chemokine RANTES [15]. The ability of a viral isolates to use one or more chimeric receptors is most probably a reflection of a more efficient usage of the CCR5 molecule, as suggested by our previous results demonstrating a higher infectivity of the wild-type CCR5 expressing cells with R5^broad^ compared to R5^narrow^ isolates [15]. In this study we asked the question whether phenotypic variation, implying different mode of CCR5 use in pregnant women could play a role in MTCT of HIV-1 and in turn, also in pediatric disease progression.

## Materials and Methods

### Patients and virus isolation

Our study population included a total of 59 HIV-1 seropositive women (24 transmitting and 35 non transmitting) and 28 infected children. Viral isolates from mother-child pairs were available in 21 cases. Additional seven children were included in the study, but the maternal samples were not available. Samples from non transmitting mothers and mother-child pairs were collected within the framework of two separate cohort studies in Northern Italy. One cohort consisted of 15 infected mother-child pairs and 17 non transmitting mothers who took part in a HIV-1 MTCT multicenter study from 1989 to 1994 [4], [6], [17], [18]. The second cohort consisted of 9 infected mother-child pairs and 18 non transmitting mothers collected in the framework of MTCT multicenter study beginning in 1986 [3], [19]. All samples were from Italian subjects and collected before the introduction of any MTCT preventive antiretroviral therapy. In 14 out of 16 children tested the infection was possibly acquired during the intrapartum period, since negative results were obtained by polymerase chain reaction (PCR) within the first week of life. Postnatal transmission was excluded in all cases, as none of the children was breast-fed. The Ethical Committee approved the use of samples according to national laws (in 1986, 1991 and 2008). An informed oral consent was obtained from the pregnant women and, in the case of the children, from the parents.

The clinical stage of the women and their children (available for 21 transmitting and 24 non transmitting mothers and for 26 out of 28 children respectively), was defined according to the guidelines of the Centers for Disease Control (CDC) [20], [21]. Most of the mothers were asymptomatic at the time of virus isolation, with the exception of three mothers: one non transmitting (clinical stage B, A206) and two transmitting mothers, one with moderate symptoms (clinical stage B, A225) and one with AIDS (clinical stage C, A130). CD4+ T cell counts were provided with the samples in many cases (for 17 transmitting and 28 non transmitting mothers) [3], [17]. HIV-1 p24 antigenemia and plasma viral load were available for a limited number of women (33 and 17 mothers, respectively) and thus, were not included in the study analysis.

HIV-1 infection of the children was determined by virus isolation and PCR [18], [22]. Clinical and immunological data of the children were obtained throughout follow-up, i.e. until death or for at least 10 years (Table 1). Children were treated only with mono or dual antiretroviral therapy (ART). Highly active ART (HAART) was not administered to children in groups 1, 2 and 3, whereas children in group 4 were treated only after entering CDC category 3 or C.

**Table 1:** Clinical and viral characteristics of HIV-1 infected children(a).

Category of progression	Child code	Phenotype(b) close to birth	Age at category diagnosed(c)			Death	Therapy(d)
			CDC 3	CDC B	CDC C		
**GROUP 1**							
**CDC 3 or early death <12 mos**	B111p	Broad (1)	4	4	-	-	60
	B117p	Narrow (5)	4	-	4	11	-
	B183	Narrow (1.5)	-	-	-	3	-
	B193	Broad (4)	6	6	12	28	12
	B196	R5X4 (1)	6	6	34	44	9
	B201	Narrow (2)	6	-	6	9	-
	B204	Broad (0)	6	6	-	38	4
	B224	Narrow (1)	6	8	44	-	8
	B314	Broad (0.5)	-	-	-	9	-
	B380	Narrow (6)	9	9	15	-	4
**GROUP 2**							
**CDC3 13–24 mos**	B252	Broad (1)	14	-	14	30	14
	B256	Narrow (1.5)	17	-	5	46	7
**GROUP 3**							
**CDC3 25–36 mos**	B32	Narrow (3)	28	28	-	96	28
	B34p	Broad (3)	28	9	-	-	70
	B130	Narrow (3)	30	1	38	47	22
	B199	Narrow (2)	27	24	-	60	27
**GROUP 4**							
**CDC3 36–60 mos**	B3	Narrow (6)	65	65	-	-	54
	B115	Narrow (3)	58	58	-	-	59
	B136	Narrow (3)	60	-	-	-	64
	B145	Narrow (1)	48	48	98	-	51
	B255	Narrow (3.5)	46	-	12	-	12
**GROUP 5**							
**CDC3 >60 mos or never**	B31p	Narrow (0)	-	-	-	-	114
	B115p	Narrow (2.5)	-	-	-	-	52
	B190	Narrow (1)	-	-	-	-	77
	B225	Narrow (1)	-	-	-	-	40
	B306	Narrow (9)	-	28	-	-	72
**GROUP 6**							
**Lost at follow-up**	B107p	Narrow (1)	n.a.	n.a.	n.a.	n.a.	n.a.
	B139p	Broad (4)	n.a.	n.a.	n.a.	n.a.	n.a.

(a) symbol - means that the event has not occurred. n.a. = not available. Mos means months of age. Age of appearance of the different conditions is always indicated in months.

(b) Narrow and broad refer to viruses with R5 phenotype. Viruses able to exclusively use wild type CCR5 receptor are defined narrow, whereas those using chimeric receptors besides the wild type CCR5 are defined broad. In parenthesis is indicated the age in months of the virus phenotype determination.

Statistical analysis: Influence of virus R5^broad^ phenotype on disease progression including children of group 1 and 2, or children of group 1, 2 and 3: p = 0.0260 and p = 0.0218 (Pearson's chi Square), respectively.

(c) Age of entry into clinical or immunological category. Categories are defined according to the Centers for Disease Controls [21]: CDC 3 = severe immune suppression; CDC B = moderate clinical manifestations; CDC C = severe clinical manifestations.

(d) Age of start of mono or dual antiretroviral therapy, not HAART.

Virus isolation was performed from patient's PBMCs as previously described [18], [22]. HIV-1 p24 antigen (Ag) positive culture supernatants, collected during first or second culture passage, were used to prepare virus stocks. Maternal viral isolates were collected during pregnancy, at delivery or within 5 months after delivery (Table 2). Virus was isolated from infected children at an age range between birth and 9 months.

**Table 2:** Comparison of viral phenotypes of 21 mother-child pairs.

Patient code(a)	Wild type coreceptor use	Chimeric receptor use	Patient code(a)	Wild type coreceptor use	Chimeric receptor use
**A107p (+1)**	R5	-	**A34p (+3)**	R5	FC 1-2-4b
**B107p (+1)**	R5	-	**B34p (+3)**	R5	FC 4b
**A115 (+3)**	R5	-	**A111p (+1)**	R5	FC 1-2
**B115 (+3)**	R5	-	**B111p (+1)**	R5	FC 1-2
**A115p (+2.5)**	R5	-	**A139p (+4)**	R5	FC 2
**B115p (+2,5)**	R5	-	**B139p (+4)**	R5	FC 4b
**A117p (+5)**	R5	-	**A145 (+1)**	R5	FC 2
**B117p (+5)**	R5	-	**B145 (+1)**	R5	-
**A130 (0)**	R5	-	**A252 (−0.5)**	R5	FC 2
**B130 (+3)**	R5	-	**B252 (+1)**	R5	FC 2-4b
**A136 (+1)**	R5	-	**A314 (0)**	R5	FC 2-4b
**B 136 (+3)**	R5	-	**B314 (+0.5)**	R5	FC 2-4b
**A224 (0)**	R5	-			
**B224 (+1)**	R5	-			
**A225 (0)**	R5	-	**A31p (0)**	R5X4	FC 1-2-4b-5-6-7
**B225 (+1)**	R5	-	**B31p (0)**	R5	-
**A255 (+0.5)**	R5	-	**A183 (0)**	R5X4	FC 2-4b-5-6-7
**B255 (+3.5)**	R5	-	**B183 (1.5)**	R5	-
**A256 (+1.5)**	R5	-	**A193 (+4)**	R5X4	FC 4b-6-7
**B256 (+1.5)**	R5	-	**B193 (+4)**	R5	FC 2-4b
			**A196 (0)**	R5X4	FC 4b-5-6-7
			**B196 (+1)**	R5X4	FC 4b-7
			**A204 (0)**	R5X4	FC 4b-7
			**B204 (0)**	R5	FC 2-4b

(a) Isolates from corresponding mother-child pairs are indicated by the same number preceded by a letter: A for mothers and B for children. Time of sampling is indicated in parenthesis as months before (-), after (+) or within 1 week from (0) delivery/birth.

Pairs were grouped according to the mother's virus phenotype, i.e. first those carrying an R5^narrow^, than an R5^broad^ and last an R5X4 virus.

Samples were used to infect U87.CD4 cells expressing wild type CCR5 or CXCR4, or one of the chimeric receptors FC-1, FC-2, FC-4b, FC-5, FC-6 or FC-7. Experiments were repeated twice. –, means no chimeric receptor is used.

### Infection of U87.CD4 cell lines expressing wild type and CCR5/CXCR4 chimeric receptors

**Figure 1:**
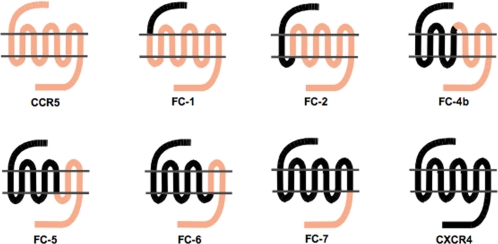
Schematic representation of the chemokine receptors CCR5 and CXCR4 and the chimeric CCR5/CXCR4 receptors. Chimeric receptors FC-1, FC-2, FC-4b, FC-5, FC-6 and FC-7 were obtained by replacing successively larger parts of CCR5 with corresponding regions of CXCR4.

Virus stocks were used to infect human glioma U87.CD4 cells stably expressing the wild type chemokine receptors CCR5 or CXCR4, or the six CCR5/CXCR4 chimeric receptors as previously described [6], [16]. Chimeric receptors were obtained by replacing, beginning from the N-terminal, successively larger parts of CCR5 with corresponding parts of CXCR4 [23]. In the resulting chimeras CXCR4 comprised gradually larger parts: the N-terminal tail only (FC-1), including the first transmembrane portion (FC-2), the first (FC-4b), second (FC-5 and FC-6) and third (FC-7) extracellular loops (Figure 1). Parental U87.CD4 cells, engineered to express CD4 but no chemokine receptor, were used as negative control.

Cells were infected in duplicate with each virus stock containing at least 2 ng/ml HIV-1 p24 Ag. The cultures were kept for 7 days and inspection for syncytia formation was performed at days 1, 3 and 7. Supernatant was collected on day 1, after washing, and at the last day of infection, and tested for the presence of p24 Ag by an in-house ELISA assay [24] (www.aaltobioreagents.ie). Cultures were only considered for evaluation if HIV-1 p24 value at day 1 was below the lower detection limit of the assay. Viral antigen production was considered positive when the absorbance at day 7 exceeded 0.2 Optical Density (O.D.).

Viruses able to use only the wild type CCR5 as coreceptor were defined as R5^narrow^, whereas R5 viruses able to use the chimeric receptors, FC-1, FC-2, and FC-4b singularly or in different combinations were defined as R5^broad^, according to the previously published classification [15]. R5X4 viruses used the six chimeric receptors in different combinations, and were not further classified.

### Statistical analysis

Correlation between R5 phenotype and immunological CDC stage of infected children were analyzed by Pearson's chi-square test. Comparison of the frequencies of the wild type chemokine receptor and chimeric receptor usage in the two groups of mothers was done by Fisher's Exact test and Pearson's chi-square test. The Mann-Whitney test and Pearson's chi-square test were used to compare the level of CD4+ T cell counts in the two groups of mothers. Analysis of variance (ANOVA) was performed to demonstrate the association between CD4+ T cell values and viral phenotype. Pearson's chi-square test was used to determine the correlation between clinical stage and the transmission status of mothers. Values below 0.05 were regarded as statistically significant.

## Results

### HIV-1 with R5^broad^ phenotype can be transmitted

Virus isolates obtained close to birth from 28 newborns were analyzed for phenotypic variability in a cell line expressing wild type or chimeric chemokine receptors. CXCR4 using virus was isolated from one newborn only, confirming previous observations that CCR5 is preferentially used by HIV-1 early after infection [3]. Twenty out of 27 children (74.07%) with an R5 virus carried a virus able to exclusively use wild type CCR5 (R5^narrow^), whereas the remaining 7 children (25.93%) harboured virus with broad use of chimeric receptors (R5^broad^) (Table 1). These results indicate that transmission of R5^broad^ virus occurred in a significant proportion of children even if the majority of viruses replicating at a time point close to infection are restricted to the use of wild type CCR5.

### The R5^broad^ phenotype is predictive of early immunological failure in children

Clinical and immunological data obtained during follow-up were available for 25 out of 27 children carrying an R5 virus close to birth and for the one child with the R5X4 virus (Table 1). The presence of viruses with R5^broad^ phenotype in the infected newborns was accompanied by a faster progression to immunological failure. Indeed, only children who experienced a severe decline of the CD4+ T cell counts as fast as within 2 or 3 years of age or died within 1 year (groups 1, 2 and 3) carried R5^broad^ viruses close to birth (p = 0.026 or p = 0.0218, Pearson's chi-square, including groups 1 and 2, or groups 1, 2 and 3, respectively). Whereas none of the children, who were classified as CDC category 3 after 36 months of age or did not enter this category during the follow up (groups 4 or 5), had a virus with R5^broad^ phenotype close to birth. The only child with a R5X4 virus showed also a fast decline of the CD4+ T cell count (classified CDC 3 within 6 months of age) and died at 44 months. Specifically, all but one R5^broad^ isolate of the newborns used FC-4b either alone (n = 2) or in combination with FC-2 (n = 4), whereas only one virus isolate used FC-1 and FC-2 (Table 2).

### Maternal viral phenotype is predictive of the newborn's virus

With the aim to understand if selective processes operate during transmission, and thus could be predictive for disease progression, we compared the phenotype of the virus isolates available from 21 mothers close to delivery with that of their newborn child (Table 2). All ten mothers harbouring an R5^narrow^ virus had children whose virus displayed the same phenotype. Interestingly, the six mothers carrying R5^broad^ viruses transmitted in all but one case a virus with R5^broad^ phenotype (identical or similar to the mother's virus); in the exceptional mother-child pair, virus with R5^narrow^ phenotype was present in the child. The five mothers with an R5X4 virus transmitted in one case R5X4, in two cases R5^broad^ and in another two cases R5^narrow^ virus. Looking at the details, coreceptor use of child's virus was narrower than the corresponding mother's virus in 7 out of 11 cases, identical in two cases, and a more flexible use of the CCR5 was observed in one child (pair 252). Thus, a virus with R5^broad^ phenotype was transmitted from the majority of mothers carrying such viruses (5 out of 6; 83.3%), indicating that the maternal viral phenotype is generally preserved during transmission and can possibly be predictive of the phenotype of the newborn's viral variant.

### Maternal viral R5 phenotype is not predictive of transmission

**Figure 2:**
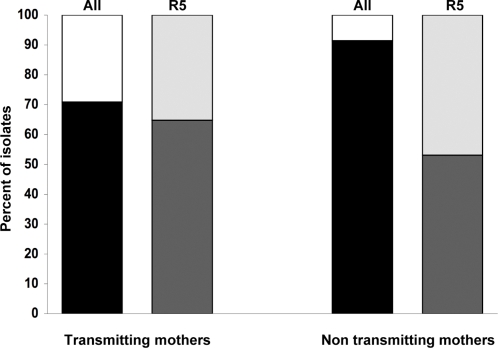
Distribution of the viral phenotype of transmitting and non transmitting mothers. Distribution of R5 (black) *vs.* R5X4 (white) viruses within all virus phenotypes (n = 59 viruses; p = n.s., Fisher's Exact Test) and of R5^narrow^ (dark gray) *vs.* R5^broad^ (light gray) within the R5 phenotype (n = 49 viruses; p = n.s., Fisher's Exact Test).

To identify factors predictive of transmission the viral phenotype of 59 mothers (24 transmitting and 35 non transmitting) was investigated. Transmitting mothers carried more often viruses able to use both CCR5 and CXCR4 as coreceptors than non transmitting mothers, 29% (7 out of 24) and 8% (3 out of 35), respectively, but this difference did not reach statistical significance (p = 0.074, Fisher's Exact Test) (Figure 2). No mother carried a monotropic X4 virus.

The analysis of the chimeric receptor usage showed that the frequency of the R5^narrow^ phenotype was similar between the two groups of mothers, 53% in non transmitting and 65% in transmitting mothers (17 out of 32 and 11 out of 17, respectively) (Figure 2). Furthermore, among the R5^broad^ isolates (15 and 6 in non transmitting and transmitting mothers, respectively), no difference was observed in the use of FC-1 and FC-2 receptors between the 2 groups of mothers, while viruses from non transmitting mothers utilized FC-4b more frequently than viruses from transmitting mothers (13 out of 15 *vs.* 2 out of 6, respectively) with a trend towards significance (p = 0.056, Perason's chi-square test). This suggests that R5^broad^ viruses from non transmitting mothers can use CCR5 at least as flexibly as viruses from transmitting mothers.

Analyses of other factors potentially correlated with risk of transmission disclosed that the two groups of mothers transmitting and non transmitting, could not be distinguished by the clinical stage (p = n.s., Pearson's chi-square test) and the CD4+ T cell counts (mean 478 and 426 cells/mm^3^; p = n.s., Mann-Whitney test). Furthermore CD4+ T cell values of mothers carrying viruses with R5^narrow^ or R5^broad^ phenotype did not differ (p = n.s., ANOVA), but were significantly different from those of mothers carrying CXCR4 using viruses (p = 0.0014, ANOVA). Thus the clinical and the immunological stage of the mothers did not seem to influence the risk of transmission in our cohort.

## Discussion

Biological characteristics of HIV-1 that are critical for the risk of MTCT continue to be subject to discussion. R5 is the predominant virus phenotype early in HIV-1 infection, both in adults and in children born to HIV-1-infected mothers. Since tools to test the mode of CCR5 use [15], [16] have been developed in the past years, we asked the question whether flexible use of CCR5, here dissected into narrow and broad phenotype, would influence transmission of HIV-1 and pediatric disease progression. In the present study the infected newborns harboured mostly viruses of R5 phenotype, a significant proportion (25.9%) of these was able to use chimeric receptors, suggesting that viral variants with a more flexible and efficient use of CCR5 (the R5^broad^ phenotype) can exist close to infection in the child.

Of utmost relevance are our data, which show that the R5^broad^ phenotype detected in the newborn shortly after birth was predictive of a fast and severe immunological failure. Thus, R5^broad^ viruses seem to determine detrimental effects similar to those known for CXCR4 using viruses. These data support the finding by Casper et al, who suggested that the immunological deterioration in HIV-1 infected children precedes the viral phenotypic switch to CXCR4 usage [25]. We suggest that pre-existing R5^broad^ viruses may have caused the worsening of the disease. Interestingly, in our study all but one newborn's R5^broad^ virus were capable of FC-4b usage, which indeed was previously shown to be linked to evolution to CXCR4 use in adults [15]. Further studies with sequential follow up samples from children will clarify if the association with CXCR4 switch occurs also in pediatric HIV-1 infection.

In neonates the memory CD4+ T cells, which express high levels of CCR5, are 6–7 times less represented than the naïve CD4+ cells [26]. The latter predominantly express CXCR4 [26], [27], [28], [29], and are primarily infected by CXCR4 using viruses [30]. It is likely, that the R5^broad^ compared to R5^narrow^ viruses may infect CD4+ naïve cells in addition to memory cells despite the limited expression of the CCR5 molecule, due to their more efficient usage of the coreceptor [15]. Interestingly, Sleasman et al. [27] described that although HIV-1 infected neonates in general contained 10 to 100 fold greater number of infected CD4+ memory cells than naïve cells, those children who rapidly progressed in the disease had high proviral load in the CD4+ naïve cells. Thus, infection of naïve cells by R5^broad^ viruses may interfere with CD4+ T cells production, and thus account for the rapid disease progression observed in children harbouring these viruses. It remains to be solved why CXCR4 using viruses are not preferentially maintained during transmission, despite the high prevalence of CXCR4+ naïve cells in neonates.

The detailed analysis of the mother's viruses in comparison to those of the corresponding child allowed us once again to pinpoint that the R5 phenotype, either narrow or broad, was usually maintained during the transmission event. On the contrary, the R5X4 phenotype was predominantly lost during transmission. Specifically, mothers with an R5X4 virus transmitted virus with a whole array of phenotypes, i.e. R5^narrow^, R5^broad^ or R5X4. These data lend further support to the lack of restriction in transmission of R5^broad^ viruses and favour the possibility that the maternal viral R5 phenotype is predictive of the transmitted variant.

Our data suggest that mothers carrying R5X4 viruses have phenotypically highly heterogenous populations. Whether a selective process or simply a random event governs transmission remains, however, a topic of discussion. [17], [31], [32], [33].

We showed that mothers harbouring R5^broad^ viruses were not at higher risk of transmission and the presence of HIV-1 with R5^broad^ phenotype was not predictive for MTCT of HIV-1. The use of chimeric receptors suggests a more flexible usage of CCR5, which in turn may indicate increased viral resistance to inhibition by CC-chemokines. In this respect, the trend of the non transmitting mothers to more often carry viruses with the specific usage of the FC-4b chimeric receptor than transmitting mothers, is intriguing. Recently, Meddows-Taylor et al. described that non transmitting mothers have significantly higher levels of CCL3 in the plasma than transmitting mothers [34]. It could be envisaged that high levels of CCL3 may be needed to efficiently inhibit R5^broad^ viruses and, as a consequence, to prevent MTCT of HIV-1. In this respect, it will be relevant to compare the level of CC-chemokines in mothers harbouring R5^broad^ and R5^narrow^ viruses as well as to study if R5^broad^ viruses have a lower sensitivity to the new CCR5 inhibiting drugs than R5^narrow^ viruses.

In summary our data show that approximately one forth of the newborn's R5 viruses have the capacity to use CCR5/CXCR4 chimeric receptors indicating that phenotypes with increased flexibility of co-receptor use are not hampered during transmission. Conversely these viral variants are significantly linked with severe immunological failure within the first years of age of the infected children. These data may have important implications for timely and appropriate therapeutic choice in pediatric HIV-1 infection.
